# Determination and Pharmacokinetics of Okanin in Rat Plasma by UltraHigh Performance Liquid Chromatography Coupled with Triple-Quadrupole Tandem Mass Spectrometry

**DOI:** 10.1155/2020/4247128

**Published:** 2020-08-28

**Authors:** Yurui Shi, Rongda Chen, Jing Xie, Li Li, Guiming Liu, Meizhu Zheng, Ning Zhang

**Affiliations:** ^1^The College of Chemistry, Changchun Normal University, Changchun 130032, China; ^2^The College of Jiamusi, Heilongjiang University of Chinese Medicine, Harbin 150040, China

## Abstract

Okanin is a major flavonoid found in *Coreopsis tinctoria* Nutt., arousing huge interest recently for its considerable biological characteristics including antioxidant, antineurotoxic, and antidiabetic activities. An ultrahigh performance liquid chromatography triple-quadrupole tandem mass spectrometry (UPLC-MS) was successfully used to determine okanin in rat plasma after oral administration of okanin. Bavachalcone acted as an internal standard (IS). By gradient elution, IS and analyte were separated on a C_18_ column for 7 min at a flow rate of 0.25 mL/min with acetonitrile-0.1% acetic acid mobile phase. The stability, matrix effect, extraction recovery, accuracy, precision, linearity, and selectivity of the method were firstly demonstrated. The major pharmacokinetic parameters of okanin in rat plasma were then measured using the developed UPLC-MS method. An UPLC-quadrupole time-of-flight mass spectrometry (UPLC-Q-TOF-MS) was finally established to obtain the specific and accurate mass of okanin in rat plasma after oral administration, and its proposed fragmentation was further elaborated.

## 1. Introduction

In China, *Coreopsis tinctoria* Nutt.'s (*C*. *tinctori*a) capitula is consumed as herbal tea, and its extracts exhibit various biological activities including antioxidant [1–4], antihypertensive [5, 6], antineuroinflammatory [7], antidiabetic activities [8–11], hepatic insulin resistance [12], and antihyperlipidemic effects [13]. Okanin refers to a major flavonoid found in *C*. *tinctori*a [14] having attracted considerable attention for its potential contribution to the pharmacological activity of *C*. *tinctori*a. Okanin had the prominent effect on DPPH scavenging acts with EC_50_ as 6.2 *μ*M, which was stronger than those of butylated hydroxytoluene and ascorbic acid with EC_50_ as 45.8 *μ*M and 30.4 *μ*M, separately [15]. Meanwhile, okanin showed a better antioxidant activity than quercetin in cellular experiments with IC_50_ as 11.0 *μ*M [15]. Kil et al. reported that okanin inhibited the production of nitric oxide and the expression of inducible nitric oxide synthetase in macrophages activated by lipopolysaccharides via nuclear factor-erythroid 2-related factor 2-dependent heme oxygenase-1 expression [16]. Furthermore, okanin could significantly inhibit *α*-glucosidase with IC_50_ values about 0.02 mM, suggesting that it is a potential drug for the treatment of diabetes [17]. Besides, okanin showed significant anti-neuroinflammatory activity with the IC_50_ value at 15.54 *μ*M, suggesting that it can treat neurodegenerative diseases [7].

Due to the significant pharmacological effects, effective methods for the identification of flavonoids in *C*. *tinctoria* become necessary. The flavonoids including okanin from *C*. *tinctoria* had been successfully analyzed by UPLC, HPLC-MS, high-performance thin-layer chromatography, and capillary zone electrophoresis methods [1, 16, 18–21]. HPLC-MS has been proved to be the most efficient analytical method for analyzing flavonoids in *C*. *tinctoria* among these methods [16, 19]. Meanwhile, it is very important to study the pharmacokinetic behavior of okanin or *C*. *tinctoria* in order to better understand their pharmacological action. Only one report emphasized the characterization of the pharmacokinetics of okanin and isookanin, the two most prominent antioxidants, in rat plasma after oral administration of 100 mg/kg ethanol extract of *C*. *tinctoria* capitula; however, the pharmacokinetic parameters of two targets only measured the time to reach *C*_max_ (*T*_max_) and the maximum plasma concentration (*C*_max_) [15]. Nevertheless, the pharmacokinetic behaviors of okanin and isookanin were not clear according to limited pharmacokinetic parameters. In the meantime, several studies have shown that the pharmacokinetic parameters of the target components were significantly different after herb extract or monomer administration, suggesting a potential pharmacokinetic interaction among multiple components in herb extracts [22–25]. Therefore, simply monitoring the pharmacokinetic parameters of target compounds in herb extracts does not represent the pharmacokinetic behavior of monomer administration. For example, the half-life of resveratrol in animal or human body was only 8–14 minutes after oral administration of monomer [22–24], but a slower elimination half-life of resveratrol was up to 5.60 h after administration of *Smilacis glabrae* extract [25]. To our knowledge, no reports have quantitatively determined okanin in rat plasma after oral monomer administration.

**Figure 1:**
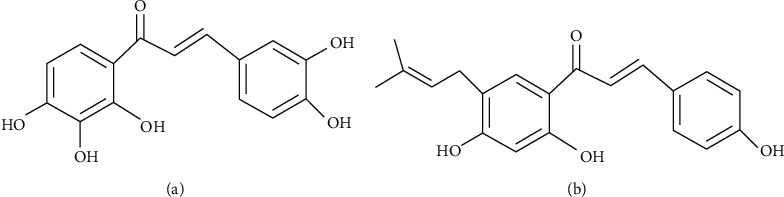
Chemical structures of (a) okanin and (b) bavachalcone.

In this work, an effective UPLC–MS/MS method coupled with multichannel segmented selected reaction monitoring program was developed for measuring the levels of okanin in rat plasma after oral monomer administration. Bavachalcone served as an internal standard (IS). The structures of the okanin and IS are shown in Figure 1. The major pharmacokinetic parameters of okanin in rat plasma were then measured using the developed UPLC-MS/MS method. The specific and accurate masses of okanin in rat plasma were finally obtained using UPLC-Q-TOF-MS, and its proposed fragmentation was further elaborated.

## 2. Experimental

### 2.1. Chemicals and Reagents

Okanin and bavachalcone with purities over 98% were provided by Shanghai Yuanye Bio-Technology Co., Ltd. (Shanghai, China). Heparin sodium was provided by Affandi (Shanghai, China). HPLC grade acetonitrile, acetic acid, and methanol were purchased from E. Merck (Darmstadt, Germany). The SPE cartridges (C_18_, 6 cc/500 mg) were provided by Wan Cheng Bo Da (Beijing, China). Triple deionized water was adopted during the study and then purified with a Millipore purification system (Bedford, MA, USA).

### 2.2. UPLC-MS/MS Conditions

An UPLC-MS/MS method was carried out using an ACQUITY^TM^ UPLC system combined with a Waters® Micromass® Quattro Premier™ XE triple-quadrupole tandem mass spectrometer analyzer (Waters Corp., Milford, MA, USA) with an electrospray ionization (ESI) interface. An ACQUITY^TM^ BEH C_18_ column (100 × 2.1 mm, 1.7 *μ*m; Waters Corp., Milford, MA, USA) combined with an ACQUITY^TM^ UPLC C_18_ guard column was used for the separation. The column temperature was set at 35°C. The binary mobile phase system was composed by 0.1% acetic acid (A) and acetonitrile (B). The gradient conditions of the mobile phase included: 0–2 min, 95–20% (A); 2-3 min, 20–20% (A); 3–3.5 min, 20–95% (A); 3.5–7 min, 95–95% (A). The flow rate was kept at 0.25 mL/min for an overall 7-min run time. The volume of sample injection was 5 *μ*L. Mass spectrometer was further improved as follows: capillary voltage, 3.50 kV (+); source temperature, 120°C; and desolvation temperature 300°C. Nitrogen was served as the desolvation and cone gas, and flow rates were set at 600 and 50 L/h, respectively. Argon acted as the collision gas at 2.89 × 10^−3^ mbar pressure. Multiple reaction monitoring (MRM) in the positive ion mode was selected in this study. The dwell time was automatically set by MassLynx NT 4.1 software. All data obtained from the centroid mode were further processed by MassLynx NT 4.1 software. The improved MRM transitions and energy parameters including cone voltage and collision energy of okanin and IS are listed in Supplementary Table 1.

The full-scan mass range was set at 50–1200 for obtaining accurate mass of okanin by UPLC-Q-TOF-MS analysis. The mass spectrometer conditions were listed as follows: gas temperature 550°C, spray gas 50 psi, auxiliary gas 50 psi, curtain gas 35 psi, ion spray voltage 5500 V, declustering potential 90 V, and collision energy for MS^2^ was 35 V.

### 2.3. Calibration Standards and Quality Control Samples

One mg/mL of okanin was prepared with methanol as standard stock solution. Then, the standard stock solution was diluted 10 times as working solution. In the meantime, one mg/mL of bavachalcone was prepared with methanol as an IS solution. Calibration standards, low, intermediate, and high levels of quality control (QC) samples were prepared using spiking blank rat plasma with appropriate volumes of the working solutions.

Six serial concentrations were 1.956, 10.95, 40.40, 200.20, 800.24, and 1390 ng/mL for calibration standard solution. The concentrations of the low, intermediate, and high levels of QC samples were 10.01, 500.10, and 1000.20 ng/mL for okanin. All samples were incubated at −20°C before application.

### 2.4. Sample Cleanup

Two hundred microlitres of calibration standards, QC samples, and plasma samples were added to a 1.5 mL Eppendorf tube containing 400 *μ*L blank plasma, and 50 *μ*L of IS solution (22.06 ng/mL) was added to respective tube.

Before loading the sample, the SPE cartridges were washed with 3 mL of methanol and 5 mL of purified water separately. The mixture in respective tube was vortexed for 1.0 min and then loaded onto a SPE cartridges. The SPE cartridges were separately eluted with 2 mL purified water and 4.5 mL of methanol. The resulting eluate was dried with nitrogen. The residue was redissolved in 50 *μ*L methanol and centrifuged at 16,000 ×*g* for 15 min. Five microlitres of aliquot was subsequently injected into the UPLC-MS for investigation.

### 2.5. Method Validation

#### 2.5.1. Specificity and Linearity of Calibration Curves, LLOD, and LLOQ

In order to study the possible endogenous interference on okanin and IS, the blank plasma from six rats, the corresponding blank plasma added with okanin and IS, and the plasma of rats after oral administration of okanin were analyzed by UPLC-MS/MS, respectively. The retention times (*R*_*t*_) and MRM transitions were adopted for analyzing okanin and IS. Meanwhile, the accurate mass of okanin and its proposed fragmentation mechanism in rat plasma after oral administration were obtained using UPLC-Q-TOF-MS.

Calibration curve for okanin was plotted for the peak area ratio of okanin/IS versus plasma concentrations by a 1/*x*^2^-weighted liner least-square regression model. The lower limit of detection (LLOD) was defined as the lowest concentration with at least three signal-to-noise ratios. The lower limit of quantification (LLOQ) was defined as the lowest concentration on the standard curve that can be quantitated with an accuracy of 80–120% and a precision value not exceeding 20%.

#### 2.5.2. Matrix Effect, Extraction Recovery, Accuracy, Precision, and Stability

Intraday and interday accuracy and precision were assessed from replicate analyses (*n* = 6) of low, middle, and high concentrations of QC samples in the same day and over three consecutive days, respectively. The concentration of sample was calculated according to the standard curve equation prepared on the same day. The precision of the method was calculated as the relative standard deviation (RSD) and was less than 15%. Accuracy was expressed as the relative error (RE) and was within ±15%. The recovery and matrix effects were measured using low-, middle- and high-concentration QC samples with six replicates. The recovery was assessed by comparing the peak areas of the spiked samples to those of the untreated sample. The matrix effect was determined by comparing the peak areas of spiked blank plasma (A) to the peak areas of the solution standards in methanol at equal concentrations (B). The matrix factor (MF) was expressed as the ratio of (A/B × 100), and the MF values ranged from 80% to 120%. The stabilities of okanin in plasma were evaluated by analyzing the three concentrations of QC samples at different storage and processing procedures. The short-term stability was also assessed by analyzing the QC sample retained in the autosampler (4°C) for 36 h. The QC sample was stored at −20°C for 30 days in order to evaluate long-term stability. The freeze-thaw stability of QC samples was evaluated by transferring them from −20°C to room temperature for three complete freeze-thaw cycles. The stability was demonstrated if the percentage deviation was no more than 15% of the nominal concentration.

### 2.6. Pharmacokinetic Analysis

The pharmacokinetics of okanin was investigated using male Sprague Dawley (SD) rats (250 ± 20 g) from Animal Safety Evaluation Center of Heilongjiang University of Chinese Medicine (China). The rats were divided into control and treatment groups with six animals each. The rats were maintained under a light/dark cycle of 12 h, in the temperature range of 22°C ± 2°C to ambient temperature, and at a humidity of 50% ± 5%. Before the experiment, all rats were fasted for 12 h, but allowed to drink water freely. Each rat in the treatment and control group were treated with 1 mg/kg okanin and an equal volume of distilled water in an oral manner, separately. Blood samples (approximately 0.4 mL) were collected into 1.5 mL heparinized polythene tubes from the suborbital venous plexus before administration and at 0, 0.083, 0.167, 0.33, 0.50, 0.75, 1.0, 2.0, 4.0, 8.0, 12.0, and 24 h after administration. Then, the blood samples were immediately centrifuged at 13,000 rpm for 10 min, and samples were processed as described in Section 2.4.

### 2.7. Data Analysis

According to observing plasma concentration versus time curve, maximum plasma concentration (*C*_max_) and the time to reach *C*_max_ (*T*_max_) were directly determined. The elimination rate constant (Kel) was then obtained via linear regression analysis on the log-linear portion of the plasma concentration versus time curve. The other major pharmacokinetic parameters of okanin in rat plasma including half life (*t*_1/2_), the mean residence time from zero to the sampling time (MRT_0–*t*_) and to infinity (MRT_0–*∞*_), and area under the plasma concentration versus time curve from zero to the sampling time (AUC_0–*t*_) and to infinity (AUC_0–∞_) were determined using WinNonlin Professional Version 5.2.1 (http://www.pharsight.com). The results were expressed as arithmetic mean ± standard deviation (mean ± SD, *n* = 6).

## 3. Results and Discussion

### 3.1. Optimization of UPLC-MS/MS Conditions

As described in Section 2.2, in order to obtain symmetric peak shape, high sensitivity, and a short run time for the separation, an accurate and reliable UPLC method for analyzing okanin and IS in rat plasma after administration had been successfully established.

Okanin and IS were separately injected into the mass spectrometer for the optimization of MS/MS conditions, and the positive ESI mode was taken for more robust and stable intensity of signal for both okanin and IS than those of the negative mode. The MRM transitions and energy parameters of okanin and IS in the positive ESI mode were further optimized, and the results are shown in Supplementary Table 1. The MRM transitions were detected at *m/z* 289.14 ⟶ 153.25 and *m/z* 325.14 ⟶ 269.19 for okanin and IS, respectively. The results indicated that the MRM model used in this study can effectively determine the target compounds in rat plasma.

### 3.2. Method Validation

#### 3.2.1. Specificity, Calibration Curve, LLOD, and LLOQ

**Figure 2:**
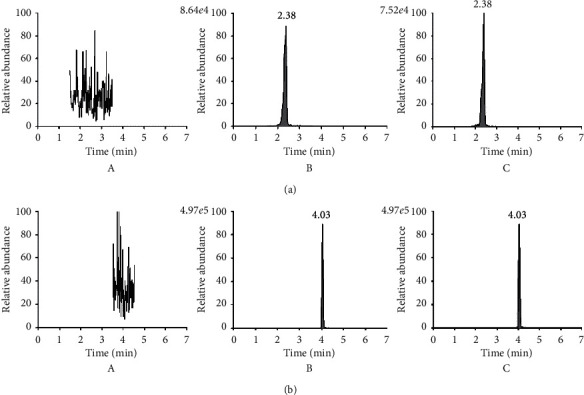
Representative MRM chromatograms of okanin and bavachalcone (IS) in positive ion mode: (a) okanin and (b) bavachalcone (IS). (A) Blank plasma. (B) Blank plasma spiked with okanin and IS. (C) Plasma sample 1 h after oral administration of okanin (1 mg/kg) (mean ± SD, *n* = 6).

Figure 2 presents typical MRM chromatograms in the positive ion mode of blank plasma, blank plasma stabbed with okanin and IS, and plasma sample 1 h after oral administration of okanin (1 mg/kg). Okanin and IS with retention times of 2.83 and 4.03 were identified by comparing retention time, precursors, and MRM transitions with those of standards. Under the UPLC-MS/MS conditions, no noticeable interfering peaks were detected overlapped with analyte elution times in blank rat plasma. Moreover, no interference appeared between IS and okanin, suggesting efficient separation of okanin and IS. The results showed that the developed method had significant specificity and selectivity for the analysis of okanin in rat plasma.

**Figure 3:**
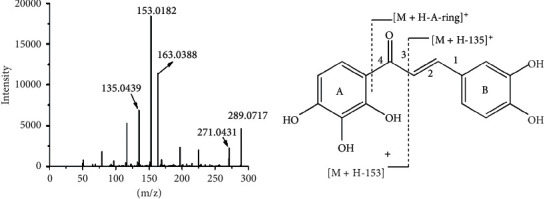
UPLC-MS^2^ Q-TOF spectra and the proposed fragmentation pathways of okanin.

Okanin in rat plasma eluted at 2.38 min showed a major molecular ion at *m/z* 289.0717 in the positive mode, suggesting the accurate mass to be 288.0717 (calculated for C_15_H_13_O_6_) by the high-resolution Q-TOF analyses for structural confirmation. In UPLC-Q-TOF-MS/MS experiment, the ion at *m/z* 289.0717 yielded four fragment ions at *m/z* 271.0431, 163.0388, 153.0182, and 135.0439, respectively. As shown in Figure 3, the ion at *m/z* 163.0388 was produced by losing A's ring. The predominant fragmentation ion at *m/z* 135.0439 and 153.0182 was formed from the cleavage of position 3 in the three-carbon chain, and the charge is retained in ring A and ring B, respectively. Minor fragment *m/z* 271.0431 was produced directly from the parent ion of *m*/*z* 289.0717 due to the neutral loss of one molecule of water, indicating the existence of orthophenol hydroxyl in the structure. The proposed fragmentation mechanism was elaborated in Figure 3.

Calibration curve was linear over the concentration ranges of 1.956–1390 ng/mL for okanin, and representative regression equation was *y* = 19.979*x* + 337.59 and *r*^2^ = 0.9980 (Supplementary Table 2). Good linearity was obtained. The LLOD and LLOQ were determined at 0.675 and 1.956 ng/mL, respectively, revealing that the presented method is sufficient for the analysis of okanin in rat plasma.

#### 3.2.2. Precision, Accuracy, Extraction Recovery, Matrix Effect, and Stability

As shown in Supplementary Table 3, the intra- and interday precision of okanin were changed by less than 5.94%, and the intra- and interday accuracy were between 2.23% to 5.97% and 1.86% to 7.40%, separately. The results revealed that the developed method was suitable for determining okanin in rat plasma.

In order to evaluate the reproducibility of the method, the extraction recovery and matrix effect experiments were carried out, and the results are listed in Supplementary Table 4. The mean extraction recoveries of okanin were greater than 101.06%, indicating that the optimized preparation was feasible and reproducible. The values of the matrix factors of three levels QC samples ranged from 97.44% to 102.46%, indicating that no coeluting endogenous substances from plasma significantly affected the ionization of the okanin. As listed in Supplementary Table 4, the stability of okanin was measured under diverse potential conditions. The results suggested the good stability of okanin over different storage conditions.

### 3.3. Application to Pharmacokinetic Study

**Figure 4:**
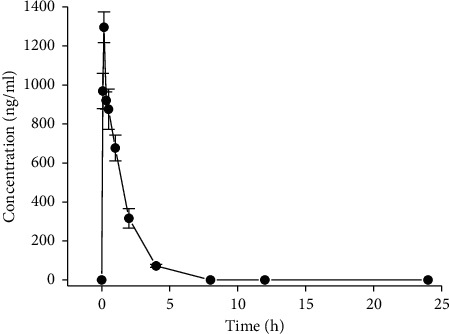
Plasma concentration vs. time profiles of okanin in rats after oral administration of okanin (1 mg/kg) (mean ± SD, *n* = 6).

Okanine in rat plasma was successfully determined by developed UPLC-MS/MS after oral administration of okanine monomer at a dose of 1 mg/kg. As shown in Figure 4, the mean plasma concentration versus time curves were plotted. After oral administration, plasma concentrations of okanin were determined for 24 h, and okanin was detected in plasma up to 8 h.

As listed in Supplementary Table 5, the major pharmacokinetic parameters were determined for control rats or rats that treated with 1 mg/kg of okanin. At a *T*_max_ of 0.167 h, the rat plasma concentration of okanin reached the maximum concentration (*C*_max_) of 1296.12 ± 60.31 ng/mL (mean ± SD), revealing quick absorption of okanin into blood circulatory system. An existing report suggested *T*_max_ and *C*_max_ values for okanin of 0.333 h and 10.5 *μ*M after oral administration of 100 mg/kg ethanol extract of *C*. *tinctoria* capitula [15]. In contrast to the literature which reported *T*_max_ values, okanin was absorbed into blood more quickly by orally administered monomer than the extract of *C*. *tinctoria*, indicating a possible pharmacokinetic interaction of multiple components in the extract of *C*. *tinctoria*. Our conclusion was consistent with that of literatures [22–25].

To further study the pharmacokinetic behaviors of okanin, more pharmacokinetic parameters including *t*_1/2_, K_el_, AUC_0–*t*_, AUC _0–∞,_ MRT_0–*t*_, and MRT_0–*∞*_ values were determined here. The concentrations of okanin in rat plasma were decreased quickly, and the shortest elimination half life (*t*_1/2_) at 0.89 ± 0.09 h was observed for okanin. The *K*_el_ values were 0.75 ± 0.08 for okanin. The AUC_0–*t*_ and AUC _0–*∞*_ values of okanin were 1728.78 ± 146.64 and 1826.51 ± 149.59 ng·h/mL, respectively. MRT_0–*t*_ and MRT_0–∞_ values for okanin were 1.14 ± 0.06 h and 1.36 ± 0.10 h, separately.

## 4. Conclusions

An effective UPLC–MS/MS method was developed for analyzing okanin in rat plasma after oral administration okanin monomer for the first time. Compared with the literature report, more pharmacokinetic parameters of okanin including *T*_max_, *C*_max_, *t*_1/2_, *K*_el_, AUC_0–*t*_, AUC _0–∞,_ MRT_0–*t*_, and MRT_0–*∞*_ values were obtained in this study. Meanwhile, target okanin had shorter *T*_max_ by monomer administration than that of herb extract administration, suggesting a potential pharmacokinetic interaction among multiple components in herb extract. The pharmacokinetic behaviors for okanin provide the basis for further studies to elucidate the mechanisms of action and facilitate clinical application.

## Data Availability

The data used to support the findings of this study are included within the article.

## Supplementary Materials

Supplementary Materials Table 1: precursor/product ion pairs and parameters for MRM of okanin used in this study. Table 2: regression equations, linearity, LLOD, and LLOQ. Table 3: intraday and interday precision and accuracy (*n* = 6). Table 4: extraction recovery, matrix effect, and stability results at low, middle, and high concentration levels (mean ± SD, *n* = 6). Table 5: the main pharmacokinetic parameters after oral administration of okanin with 1 mg/kg (mean ± SD; *n* = 6).
