# Supplementary material for: Determination and Pharmacokinetics of Okanin in Rat Plasma by UltraHigh Performance Liquid Chromatography Coupled with Triple-Quadrupole Tandem Mass Spectrometry
Source: J Anal Methods Chem. 2020 Aug 28;2020:4247128. doi: 10.1155/2020/4247128 (PMC7474779; doi:10.1155/2020/4247128)
Supplement: Supplementary Materials — Table 1: precursor/product ion pairs and parameters for MRM of okanin used in this study. Table 2: regression equations, linearity, LLOD, and LLOQ. Table 3: intraday and interday precision and accuracy (n = 6). Table 4: extraction recovery, matrix effect, and stability results at low, middle, and high concentration levels (mean ± SD, n = 6). Table 5: the main pharmacokinetic parameters after oral administration of okanin with 1 mg/kg (mean ± SD; n = 6). [file 4247128.f1.docx]

**Table 1.** Precursor/product ion pairs and parameters for MRM of okanin used in this study.

| Analytes | R_t_(min) | Precursor  [M+H]+ | MRM transitions (precursory→product) | Cone  (V) | Collision  (V) |
| --- | --- | --- | --- | --- | --- |
| Okanin | 2.38 | 289.14 | 289.14→153.25 | 30 | 22 |
| Bavachalcone | 4.03 | 325.14 | 325.14→269.19 | 25 | 25 |

**Table 2.** Regression equations, linearity, LLOD and LLOQ

| Analytes | Regression equation (r^2^) | Linear range (ng/mL) | LLOD (ng/mL) | LLOQ (ng/mL) |
| --- | --- | --- | --- | --- |
| Okanin | Y=19.979X+337.59（0.998） | 1.956-1390 | 0.675 | 1.956 |

**Table 3.** Intra-day and inter-day precision and accuracy (n=6)

| Components | Concentration  added (ng/mL) | Inter–day concentration  measured (ng/mL) | Precision  (%,RSD) | Accuracy  (%,RE) | Intra-day concentration  measured (ng/mL) | Precision  (%,RSD) | Accuracy  (%,RE) |
| --- | --- | --- | --- | --- | --- | --- | --- |
|  | 10.01 | 10.29 ± 0.57 | 5.54 | 1.86 | 10.33 ± 0.57 | 5.55 | 2.23 |
| Okanin | 500.10 | 537.13 ± 24.21 | 4.51 | 7.40 | 529.97 ± 23.10 | 4.36 | 5.97 |
|  | 1000.20 | 1044.20 ± 59.24 | 5.67 | 4.40 | 1041.80 ± 61.92 | 5.94 | 4.16 |

**Table 4.** Extraction recovery, matrix effect and stability results at low, middle and high concentration levels (mean±SD, n=6)

| Component | Spiked (ng/mL） | Extraction recovery(%) | Matrix effect  (%) | Auto-sampler stability  (4°C, 36 h ) | | Long-term Stability  (-20°C, 30 days ) | | Freeze-thaw Stability  (-20°C -RT) | |
| --- | --- | --- | --- | --- | --- | --- | --- | --- | --- |
|  |  |  |  | Measured (ng/mL） | RE（%） | Measured (ng/mL） | RE（%） | Measured (ng/mL） | RE（%） |
| Okanin | 10.01 | 101.92±7.54 | 102.46±4.80 | 9.76±0.69 | -2.98 | 10.50±0.63 | 3.98 | 10.55±0.74 | -3.81 |
|  | 500.10 | 101.69±8.32 | 101.79±6.26 | 536.31±40.87 | 0.49 | 546.24±34.09 | 9.22 | 542.04±30.73 | -1.61 |
|  | 1000.20 | 101.06±5.73 | 97.44±3.44 | 1026.37±78.27 | 2.24 | 1040.79±56.08 | 4.06 | 1054.01±44.23 | 5.38 |

**Table 5.** The main pharmacokinetic parameters after oral administration of okanin with 1 mg/kg (mean±SD; n=6)

| Analytes | T_max_ (h) | C_max_ (ng/mL) | K_el_ | T_1/2_ (h) | AUC_0-t_(ng.h/mL) | AUC_0-∞_ (ng.h/mL) | MRT_0-t_ (h) | MRT_0-∞_ (h) |
| --- | --- | --- | --- | --- | --- | --- | --- | --- |
| Okanin | 0.167±0.00 | 1296.12±60.31 | 0.75±0.08 | 0.89±0.09 | 1728.78±146.64 | 1826.51±149.59 | 1.14±0.06 | 1.36±0.10 |
